# Beyond vasodilation: reframing inhaled nitric oxide as a precision therapy

**DOI:** 10.1093/ajrccm/aamag313

**Published:** 2026-06-19

**Authors:** Chong Lei, Nikolay O Kamenshchikov, Sylvia Ranjeva, Run Dong, Pankaj Arora, Lorenzo Berra

**Affiliations:** Department of Anesthesiology and Perioperative Medicine, Xijing Hospital, Xi’an, China; Key Laboratory of Anesthesiology (The Fourth Military Medical University), Ministry of Education of China, Xi’an, China; Anesthesia Clinical Research Center, Xijing Hospital, Xi’an, China; Cardiology Research Institute, Tomsk National Research Medical Center, Siberian State Medical University, Russian Academy of Sciences, Tomsk, Russia; Department of Anesthesia, Critical Care and Pain Medicine, Beth Israel Deaconess Medical Center, Boston, MA, United States; Harvard Medical School, Boston, MA, United States; Harvard Medical School, Boston, MA, United States; Department of Anesthesia, Mass General Brigham, Boston, MA, United States; Anesthesia Center for Critical Care Research, Massachusetts General Hospital, Boston, MA, United States; Medical ICU, State Key Laboratory of Complex Severe and Rare Diseases, Peking Union Medical College Hospital, Peking Union Medical College & Chinese Academy of Medical Sciences, Beijing, China; Division of Cardiovascular Disease, University of Alabama at Birmingham, Birmingham, AL, United States; Harvard Medical School, Boston, MA, United States; Department of Anesthesia, Mass General Brigham, Boston, MA, United States; Anesthesia Center for Critical Care Research, Massachusetts General Hospital, Boston, MA, United States

Since its initial US Food and Drug Administration (FDA) approval in 1999, inhaled nitric oxide (iNO) has been indicated exclusively for term and near-term neonates with hypoxic respiratory failure and pulmonary hypertension. Potential applications in acute respiratory distress syndrome (ARDS), cardiac surgery, transplantation, right heart failure, infection, and extracorporeal membrane ­oxygenation (ECMO) remain outside this regulatory boundary. This constraint has shaped both clinical perception and research: iNO is still commonly framed as a *selective pulmonary vasodilator* that improves ventilation-perfusion matching and oxygenation. Although clinically accurate, this description is biologically incomplete for a molecule with complex redox, immunologic, antimicrobial, mitochondrial, and cardiovascular effects.

This perspective does not revisit iNO as a rescue vasodilator or propose incremental clinical indications. Rather, we argue for a conceptual shift: iNO should be studied and applied as a measurable, dose-flux–dependent, and biologically targeted therapy that may require patient selection, exposure assessment, and response stratification. A key methodological problem in prior ­trials is the implicit equivalence of delivered concentration with therapeutic exposure.

This narrowed framing has informed decades of clinical trials. We repeatedly tested whether a fixed low concentration of NO improves outcomes across heterogeneous pulmonary diseases and systemic syndromes, often yielding inconsistent results.^1-5^ The prevailing conclusion—iNO improves oxygenation but not survival—may therefore reflect limitations in questions and ­methods more than limitations of the molecule.

The reality is that we have emphasized administered concentration rather than biological uptake and target delivery. In pharmacology, dosing without measuring drug exposure overlooks pharmacokinetic and pharmacodynamic variability. Similarly, iNO is prescribed in parts per million (ppm), whereas absorption and downstream bioactivity are rarely quantified. Regulatory frameworks define gas delivery, but therapeutic effect depends on changing pathobiology. The historical inability to quantify NO bioavailability has been a measurement limitation, not an inherent limitation of NO itself. Emerging bedside methodologies now enable estimation of NO uptake and downstream biological effects.

Nitric oxide is highly reactive; within milliseconds it interacts with Hb, oxidative stress, endothelial injury, proteins, and metal ions. Clinical effects depend on the fraction of biologically meaningful reactions, not simply on ppm of the ventilator. Pulmonary uptake varies with gas flow, blood flow, FRC, membrane integrity, redox gradients, and scavenging, most of which are unmeasured at the bedside.^6^ Rather than an obstacle, this complexity argues for biologically informed dosing and phenotyping, consistent with precision medicine approaches in ARDS and sepsis.^7^

Cardiopulmonary bypass illustrates why context matters. Mechanical hemolysis releases plasma Hb that avidly consumes endogenous NO, impairing microvascular perfusion and contributing to acute kidney injury. In this biologically defined state of NO depletion, perioperative NO supplementation reduced kidney injury and improved renal recovery.^3^^,^^4^ However, when similar therapy was applied to broader endothelial dysfunction with less hemolysis-mediated NO scavenging, the benefit was not reproduced.^5^ These findings appear discordant only if NO is viewed as a uniform exogenous therapy; interpreted as supplementation of patient- and organ-specific NO bioavailability, they are coherent.

A similar pattern is observed in ARDS. The iNO reliably improves oxygenation across diverse populations,^1^^,^^2^ yet definitive survival benefit remains difficult to demonstrate. This likely reflects biological heterogeneity; distinct pathophysiological processes may require different therapies. Inhaled nitric oxide primarily targets ventilation-perfusion mismatch, whereas prone positioning and positive end-expiratory pressure address ventilation heterogeneity and recruitability. Recent analyses also suggest variability in NO responsiveness across patient subsets, consistent with heterogeneity in endogenous NO pathways.^8^

These observations motivate practical questions that can be operationalized in trial design: How should a biological responder be defined beyond transient oxygenation improvement? Which disruption states (eg, hemolysis-mediated scavenging, microvascular dysfunction, redox imbalance) predict benefit? What absorbed dose, rather than delivered concentration, restores bioavailability? And, which physiological or biochemical markers confirm target engagement? They also force reconsideration of timing (prevention vs rescue) and of exposure definition during ECMO.

Answering these questions requires endpoints beyond oxygenation. Improved oxygenation is a physiological signal, not proof of disease modification. If NO is intended to reverse hemolysis-driven microvascular dysfunction, modulate platelet activation, or exert antimicrobial nitrosative stress, these processes should be measured directly. Delivery of NO should integrate (1) quantification of NO uptake or metabolites rather than delivered concentration; (2) identification of biological states of NO disruption; and (3) dose-response relationships based on NO flux and mechanistic effect (Figure 1).

**Figure 1:**
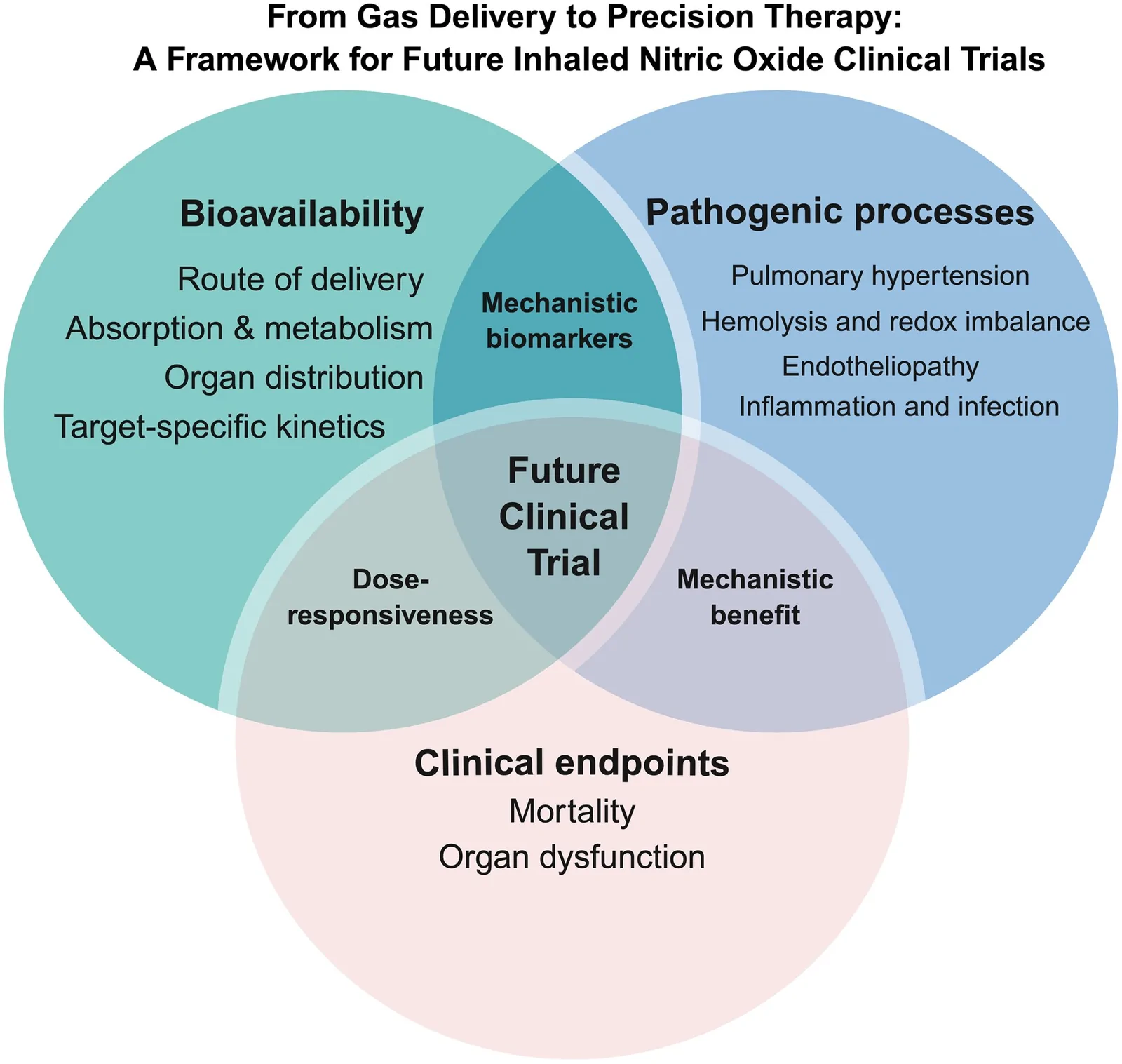
From gas delivery to precision therapy in inhaled nitric oxide. Future clinical trials of inhaled nitric oxide should integrate 3 domains: (1) drug bioavailability, including delivery route, absorption, metabolism, organ distribution, and target-specific kinetics; (2) the pathogenic processes disrupting nitric oxide signaling; and (3) clinically meaningful outcomes. Their intersections define dose responsiveness, mechanistic biomarkers, and mechanistic benefit, enabling biologically informed trial design.

Recent work illustrates how changing the hypothesis and dosing strategy changes what NO can do. In a translational study, iNO was titrated to achieve bactericidal activity: intermittent delivery at 300 ppm functioned as a nitrosative antimicrobial, reduced multidrug-resistant bacterial burden, and was feasible in humans.^9^ The molecule was unchanged; exposure targets and endpoints were different.

These principles are particularly relevant for emerging ECMO applications. Adding NO to ECMO circuits to reduce platelet activation and prolong circuit life is biologically plausible. Yet 20 ppm into the circuit is not a uniform exposure; delivery depends on membrane properties, sweep gas and blood flows, hemolysis, and redox state. Without measuring absorption and downstream biological signals (platelet activation markers, NO metabolites, hemolysis indices), we risk repeating the same mistake: equating delivered concentration with effective therapy. The evidence may not indicate that NO failed, but that we asked it to behave as if it were simpler than it is.

Critical care has already moved beyond fixed-dose thinking. Oxygen is titrated, ventilation individualized, and perfusion monitored. Inhaled nitric oxide should follow the same trajectory. Reframing iNO as a context-dependent biologic intervention would reshape study design and clinical application. Trials should enrich for defined states of NO disruption (hemolysis, endothelial dysfunction, and pathogen-driven redox imbalance) rather than undifferentiated syndromes; define dose by absorbed flux within diseased tissue; and develop biomarkers that distinguish responders from nonresponders while measuring NO bioavailability rather than assuming it.

Decades of mixed results have nonetheless revealed 2 realities: disease heterogeneity and complexity of NO biology. Recognizing these is not a failure but an opportunity. Mechanistic and translational studies enable a more mature chapter of NO therapeutics, one in which delivery, biology, and patient response are quantified. As understanding deepens, clinical meaningful indications for NO will expand.

Author contributions

Chong Lei and Lorenzo Berra wrote the first draft of the editorial. Nikolay O. Kamenshchiko, Sylvia Ranjeva, Run Dong, and Pankaj Arora revised and agreed on the final draft.

## Supplementary Material

aamag313_Supplementary_Data
